# Targeting SLC5A2 suppresses colorectal tumour development by enhancing NK cell activity through extracellular vesicle‐dependent MICA/B signalling

**DOI:** 10.1002/ctm2.70657

**Published:** 2026-04-12

**Authors:** Jun Xiao, Jianghua Wu, Fengliu Deng, Chaoqun Liu, Yuanhang Chen, Ke Shen, Chuangyuan Wang, Wandie Lin, Weiwei Liu, Ziyan Ning, Rui Zhou, Liang Zhao

**Affiliations:** ^1^ Department of Pathology Nanfang Hospital Southern Medical University Guangzhou China; ^2^ Department of Urology The Fifth Affiliated Hospital of Southern Medical University Guangzhou China; ^3^ Department of Pathology & Guangdong Province Key Laboratory of Molecular Tumor Pathology, School of Basic Medical Science Southern Medical University Guangzhou China; ^4^ Department of Pathology &The Third Xiangya Hospital of Central South University The Third Xiangya Hospital of Central South University Hunan China; ^5^ Department of Oncology Xingyi People's Hospital Guizhou China; ^6^ Department of Pathology The Eighth Affiliated Hospital of Southern Medical University (The First People's Hospital of Shunde, Foshan) Foshan China; ^7^ Department of Neurosurgery, Zhujiang Hospital Southern Medical University Guangzhou China; ^8^ Department of Pathology Guangdong Provincial People's Hospital (Guangdong Academy of Medical Sciences) Southern Medical University Guangzhou Guangdong China

**Keywords:** colorectal cancer, dapagliflozin, extracellular vesicle, MHC class I molecules, sodium‒glucose cotransporter 2

## Abstract

Tumour metabolic modulation represents a promising adjuvant therapeutic strategy for cancers, including colorectal cancer (CRC). Dapagliflozin, a clinically approved sodium‒glucose cotransporter 2 (SLC5A2/SGLT2) inhibitor, has attracted considerable attention, yet its functional role in CRC remains unclear. Here, we investigated the oncogenic effect of SLC5A2 on the colorectal mucosal epithelium using transgenic rats and an azoxymethane/dextran sulphate sodium (AOM/DSS)‐induced tumour model. Multiple immunofluorescence and tissue microarray analyses of clinical samples revealed an inverse correlation between SLC5A2 expression and natural killer (NK) cell infiltration, highlighting the therapeutic potential of dapagliflozin for CRC treatment. Mechanistically, gene expression profiling analysis and coculture experiments demonstrated that SLC5A2 impairs NKG2D‐mediated NK cell cytotoxicity. Furthermore, perforated patch‒clamp and calcium imaging revealed that SLC5A2 modulates the membrane potential and calcium influx, enhancing MHC‐I‐associated MICA/B secretion via extracellular vesicle (EV) formation and thereby enabling CRC cells to evade NK cell surveillance. Our findings reveal a critical oncogenic role of SLC5A2 in CRC progression and suggest dapagliflozin as a novel therapeutic option, particularly for CRC patients with metabolic comorbidities.

## Graphical abstract

SLC5A2 modulates the membrane potential and calcium influx, enhancing MHC‐I‐associated MICA/B secretion via extracellular vesicle (EV) formation and thereby enabling CRC cells to evade NK cell surveillance. Our findings reveal the critical role of SLC5A2 in CRC progression and suggest dapagliflozin, a clinically approved sodium‒glucose cotransporter 2 (SLC5A2/SGLT2) inhibitor, as a novel therapeutic option, particularly for CRC patients with metabolic comorbidities.

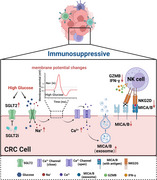

## 1 INTRODUCTION

Colorectal cancer (CRC) is one of the most prevalent malignant neoplasms worldwide. The tumour microenvironment (TME) significantly influences the progression, therapeutic response, and clinical outcomes of CRC.^1^ The metabolic processes of tumour cells rely on the microenvironment, and in turn, tumour cells substantially alter the characteristics of the TME by consuming nutrients to sustain their survival and proliferation.^2^ Increased glucose metabolism is a hallmark of many tumours; beyond the well‐characterised Warburg effect, dysregulation of glucose metabolism can modulate the quantity and activity of immune cells, impair both innate and adaptive immunity, and facilitate immune evasion by tumour cells.^3^, ^4^ In recent years, the advent of targeted therapies and immunotherapies has somewhat mitigated disease progression in patients with CRC.^5^, ^6^ Nevertheless, the rapid disease progression and the limited infiltration of immune cells considerably restrict the efficacy of these treatments. Therefore, investigating the key regulatory molecules within the TME of CRC is of paramount importance for advancing clinical treatment strategies.

Abnormal glucose metabolism is highly prevalent in CRC patients, and Type 2 diabetes is recognised as a significant risk factor for CRC.^7^, ^8^, ^9^ Current research indicates that metformin and GLP‐1 receptor agonists exert inhibitory effects on CRC.^10^, ^11^ Historically, antidiabetic drugs were believed to influence tumour progression solely through the regulation of blood glucose levels. However, recent research has challenged this traditional view, demonstrating that antidiabetic drugs can also exert antitumour effects by remodelling the TME.^12^, ^13^, ^14^ Dapagliflozin, a selective inhibitor of SLC5A2, reduces blood glucose independently of insulin by inhibiting glucose reabsorption in the renal tubules and promoting urinary glucose excretion. It was approved by the US FDA in 2014 for the treatment of type 2 diabetes.^15^ Beyond their role as antidiabetic drugs, increasing evidence indicates that SLC5A2 inhibitors confer protective effects on metabolic reprogramming in the heart, kidneys, and liver^16^, ^17^, ^18^ and are extensively utilised in clinical practice. However, their direct effects on CRC remain to be elucidated.

Therefore, in the present study, we engineered a transgenic rat model combined with an in vivo tumour induction protocol to investigate the association between SLC5A2 and CRC development. Through tumour transcriptome sequencing and functional validation, our findings revealed that SLC5A2 mediates CRC immune escape by influencing ligand‒receptor interactions. These results elucidate the potential contribution of SLC5A2 to the modulation of the CRC microenvironment and highlight the novel clinical application of dapagliflozin as a targeted therapeutic agent for SLC5A2.

## 2 RESULTS

### 2.1 SLC5A2 promotes the tumourigenesis and development of CRC in vivo

**FIGURE 1:**
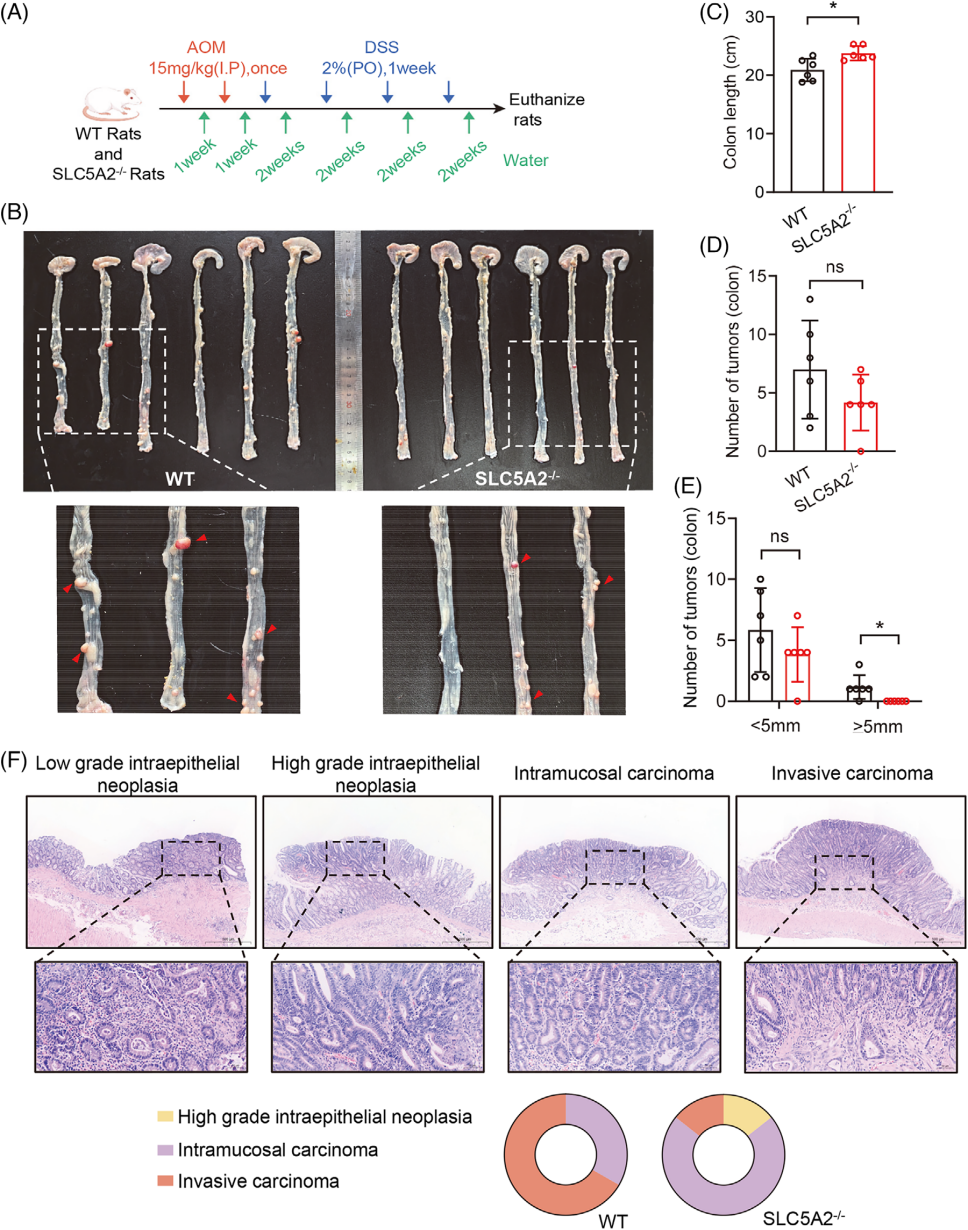
SLC5A2 promotes the tumourigenesis and development of CRC in vivo. (A) Schematic diagram of the AOM/DSS‐induced CRC rat model. (B) Macroscopic images of colons from each group treated with AOM/DSS. (C–E) Statistics of colon length, tumour number, and tumour load derived from WT (*n* = 6) and SLC5A2^−/−^ (*n* = 6) rats treated with AOM/DSS. (F) Representative HE images of colorectal tumours at different stages and the proportions of each group (*n* = 6).

We conducted a comparative analysis of genes associated with abnormal glucose metabolism and CRC, identifying SLC5A2 as a common promoting factor (Figure S1). To elucidate the role of SLC5A2 in CRC development, we constructed SLC5A2 knockout SD rats (SLC5A2^−/−^) and used wild‐type SD rats (WT) as controls (Figure S2A and B). The CRC model was established via combined treatment with azoxymethane (AOM) and dextran sulphate sodium (DSS) (Figure 1A). Upon observing the presence of bloody stools, diarrhoea, weight loss and other symptoms in the rats, the model was deemed successful. The rats were subsequently dissected, and macroscopic observations revealed that the tumours were predominantly located in the colon and rectum and were characterised by polypoid protrusions into the intestinal cavity (Figure 1B). Histological analysis showed that the tumour cells formed adenomatous structures of varying sizes and irregular shapes which infiltrated the submucosa by penetrating the colorectal mucosal muscle without invading the muscularis propria. Surrounding the tumour and normal mucosa, we observed crypt dilatation, inflammatory cell infiltration and fibrous tissue hyperplasia, submucosal oedema, connective tissue hyperplasia, increased number of blood vessels, and enlarged lumen of the proliferative blood vessels, and inflammatory cell infiltration (Figure 1F). Statistical analysis showed no significant difference in tumour number between the two groups. However, compared with the SLC5A2^−/−^ group, the WT group exhibited shorter colon length, larger tumour volume, and more severe tumour invasion (Figures 1C–F and S2C). These findings suggest that SLC5A2 knockout inhibits CRC progression in vivo.

### 2.2 SLC5A2 promotes CRC progression by influencing the TME

**FIGURE 2:**
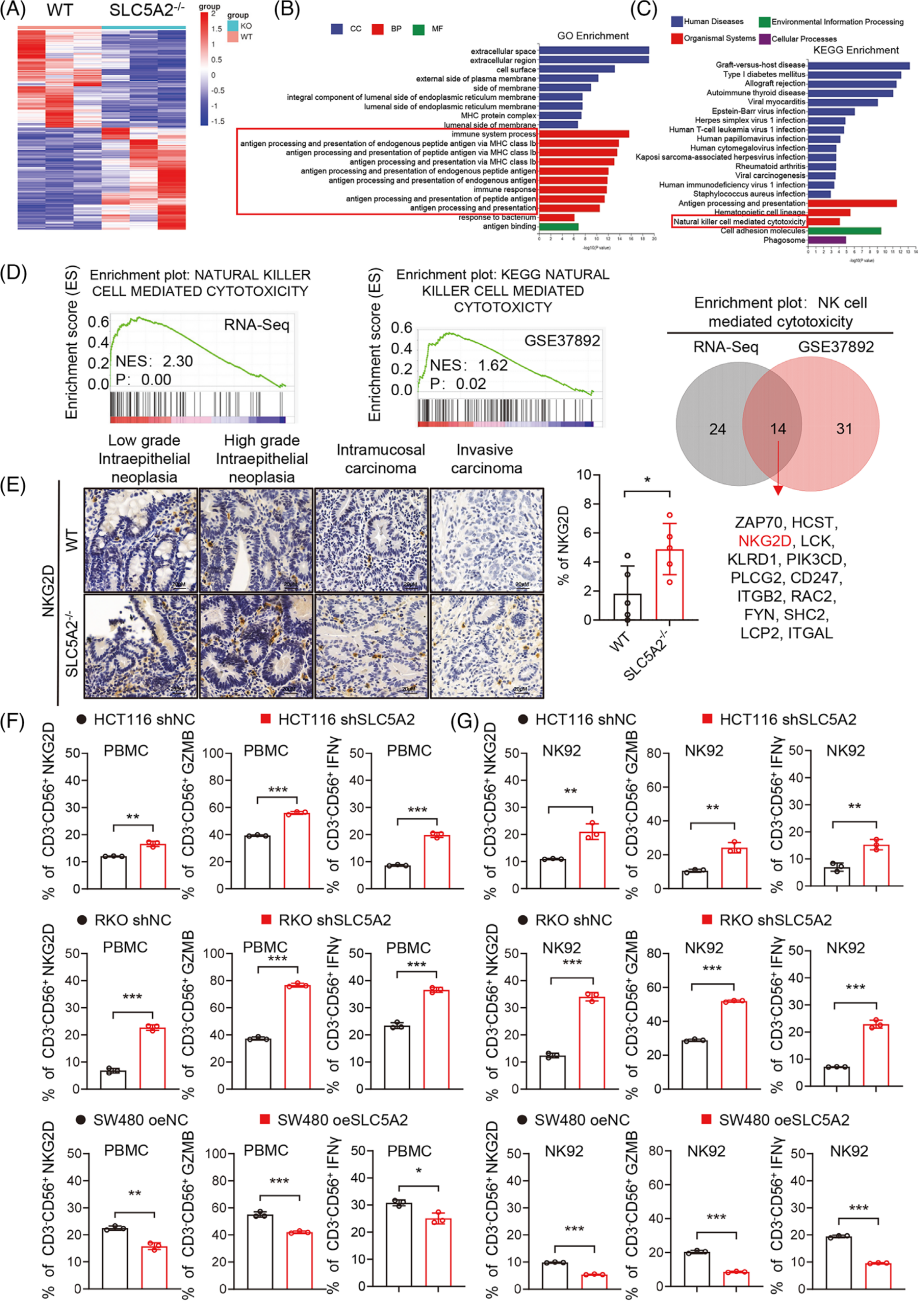
SLC5A2 inhibits NK‐cell activation by downregulating NKG2D. (A) RNA‐seq heatmaps of the WT group (*n* = 3) and SLC5A2 group (*n* = 3). (B, C) GO and KEGG analyses were performed to reveal the functions of the differentially expressed genes. (D) NK‐cell core genes associated with SLC5A2 expression in rat and human GSEA enrichment analysis. (E) The expression levels of NKG2D in tumours from the WT and SLC5A2^−/−^ groups at different stages of CRC were detected by IHC. (F, G) The percentages of NKG2D^+^, GZMB^+^, and IFN‐γ^+^ cells among CD3^−^CD56^+^ NK cells from PBMCs and NK92 cells cocultured with CRC cells were detected by flow cytometry.

To clarify the underlying mechanism by which SLCSA2 promotes CRC progression in vivo, we performed RNA‐seq on fresh SD rat tumour tissues. Transcriptome analysis revealed that SLC5A2 mainly regulated MHC‐I antigen processing, endogenous peptide presentation, and natural killer (NK) cell‐mediated cytotoxicity (Figure 2A–C). These findings imply that SLC5A2 may mediate crosstalk between CRC cells and NK cells. In addition, analysis of the human CRC GEO dataset (GSE37892) demonstrated that NK cell pathway activation was upregulated in the group with low SLC5A2 expression, corroborating the results obtained from tumour tissue sequencing. By comparing differentially expressed molecules and biological functions across the two datasets, we initially identified the human MHC‐I antigen‐associated NK cell‐activating receptor NKG2D (KLRK1) as the key receptor mediating SLC5A2‐regulated NK cell activation (Figures 2D, S3A and B). To verify the sequencing results, immunohistochemical analysis of NKG2D in rat CRC tumour tissues at different stages revealed a significant upregulation of NKG2D‐expressing cells in the SLC5A2^−/−^ group compared with the WT group (Figure 2E). Based on published literature, CD161 is a well‐recognised specific marker for rat NK cells. Thus, we performed IHC staining for CD161 in WT and SLC5A2^−^/^−^ rat tumour, which showed that SLC5A2 knockdown promoted NK cell infiltration (Figure S8A). No significant differences were detected in the numbers of cancer‐associated fibroblasts (CAFs), CD8^+^ T cells or macrophages between the two groups (Figure S4).

To further investigate the regulatory role of SLC5A2 in NK cells, we silenced SLC5A2 expression with shRNAs in HCT116 and RKO cell lines and overexpressed SLC5A2 in SW480 cell line. SLC5A2‐shRNA1, which exhibited increased silencing efficiency, was selected for subsequent experiments (Figure S5A and B). In vitro analyses indicated that SLC5A2 does not directly affect the proliferation or migration of CRC cells (Figure S5C and D). We subsequently directly cocultured human CRC cells with PBMCs isolated from the blood of healthy adults and the NK92 cell line. Compared with the control group, the silenced group exhibited significantly upregulated expression of the membrane surface receptor NKG2D and increased secretion of granzyme B (GZMB) and interferon‐γ (IFNγ) in CD3^−^CD56^+^ NK cells were significantly upregulated in the silenced group, whereas the opposite effects were observed in the overexpression group (Figures 2F and G and S6A and B). These results suggest that SLC5A2 may inhibits NK cell activation by downregulating NKG2D and hindering NK cell‐mediated cytotoxicity in CRC cells.

### 2.3 SLC5A2 promotes calcium influx‐mediated EV formation in CRC cells to escape NK cell killing

**FIGURE 3:**
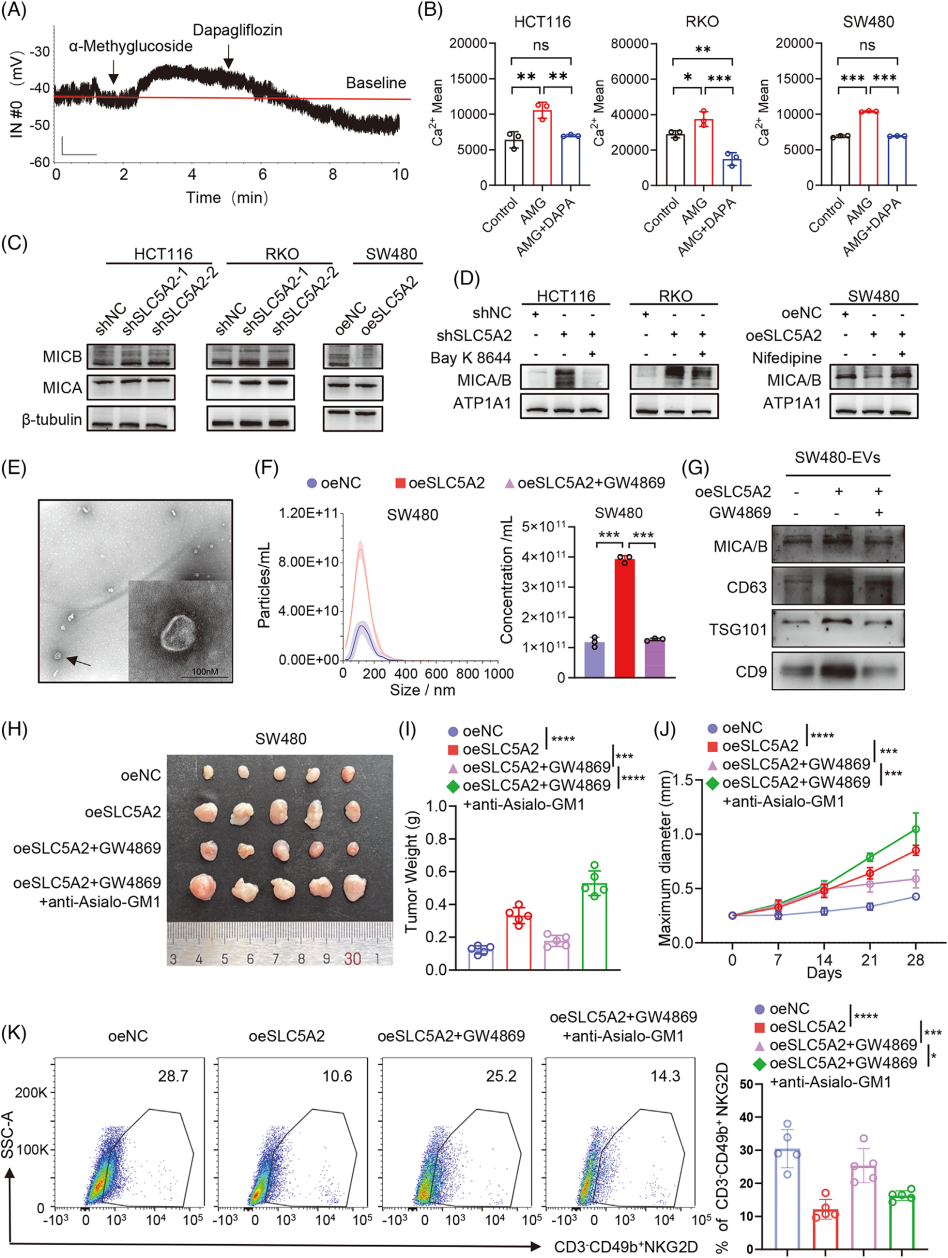
SLC5A2 promotes calcium influx‐mediated EV formation in CRC cells to escape NK cell killing. (A) Changes in the membrane potential were recorded using a perforated patch clamp. (B) The effects of SLC5A2 agonists and inhibitors on intracellular calcium in CRC cells were detected by flow cytometry. (C) Western blot analysis of the expression levels of MICA and MICB in each group. (D) Western blot analysis of the expression levels of MICA/B on the cell surface of each group in response to calcium ion agonists and inhibitors. (E) Transmission electron microscopy (TEM) analysis of the EVs secreted by SW480 cells. (F) Measurement of the particle sizes of the vesicles secreted from each group by nanoparticle tracking analysis (NTA). (G) Western blot analysis of EV markers and MICA/B extracted from equal numbers of different treatment groups of cells. (H) Effects of combined treatment with SLC5A2 overexpression, the exosome inhibitor GW4869, and the NK‐cell scavenger anti‐Asialo‐GM1 on subcutaneous tumours in nude mice (*n* = 5). (I, J) Quantification of tumour weight (I) and tumour growth curve (J) (*n* = 5). (K) The percentages of NKG2D^+^ cells among CD3^−^CD49b^+^ NK cells from subcutaneous tumours in nude mice were detected by flow cytometry.

Recent studies have highlighted the significant role of dapagliflozin in cardiovascular therapy, with its mechanism linked to alterations in calcium currents within cardiomyocytes induced by dapagliflozin.^19^, ^20^, ^21^ These findings prompted an investigation into whether SLC5A2, a sodium‒glucose transport channel, induces similar changes in CRC cells. The addition of α‐methylglucoside (AMG) resulted in membrane depolarisation and an increase in the concentration of intracellular calcium ions. Conversely, the introduction of dapagliflozin led to membrane repolarisation and a decrease in intracellular Ca^2+^ levels (Figure 3A and B). These results indicate that SLC5A2 modulation alters membrane potential and regulates calcium influx in human CRC cells.

NKG2D is an activating receptor on the surface of NK cells. Among its ligands, the MIC family has been identified as an independent prognostic marker for CRC, and its expression is associated with innate immune cell infiltration.^22^ Consequently, we detected the expression levels of MICA and MICB in CRC cells following SLC5A2 silencing or overexpression. Compared with those in the negative control group, the SLC5A2‐silenced group increased protein levels of MICA and MICB, whereas the opposite effects were observed in the SLC5A2‐overexpressing group (Figure 3C). Furthermore, treatment with Bay K 8644 (a calcium agonist) and nifedipine (a calcium inhibitor) and discovered that both agents mitigated the impact of SLC5A2 on MICA/B expression on the cell membrane surface (Figure 3D). Our findings indicate that SLC5A2 may suppress MICA/B expression on tumour cell membrane by facilitating Ca^2+^ influx.

Tumour‐derived extracellular vesicles (EVs) are key crucial mediators of crosstalk between tumour cells and the TME. Studies have shown that increased intracellular Ca^2+^ concentration can promote the release of PD‐L1 via EVs.^23^ To test whether this mechanism applies to MICA/B, we analysed the CRC cell supernatant and confirmed the presence of EVs containing MICA/B. Notably, SLC5A2 overexpression increased the secretion of MICA/B EVs in the cell supernatant, and treatment with the EV inhibitor GW4869 effectively suppressed this release (Figure 3E–G). To investigate whether SLC5A2 influences NK cell infiltration in the TME by regulating MICA/B containing EV secretion, we established a subcutaneous xenograft model in nude mice. SLC5A2 overexpression increased the tumour burden, promoted tumour progression and inhibited NK cell infiltration, and these effects were reversed by GW4869 treatment. Furthermore, combined treatment with anti‐Asialo‐GM1 abrogated MICA/B‐mediated NK cell cytotoxicity (Figures 3H–K and S8B). These results indicate that SLC5A2 contributes to CRC progression by modulating the membrane potential to induce Ca^2+^ influx, thereby promoting the secretion of EVs containing MICA/B and the evasion of NK cell‐mediated cytotoxicity.

### 2.4 Dapagliflozin inhibits CRC tumourigenesis and progression CRC by promoting NK cell infiltration

**FIGURE 4:**
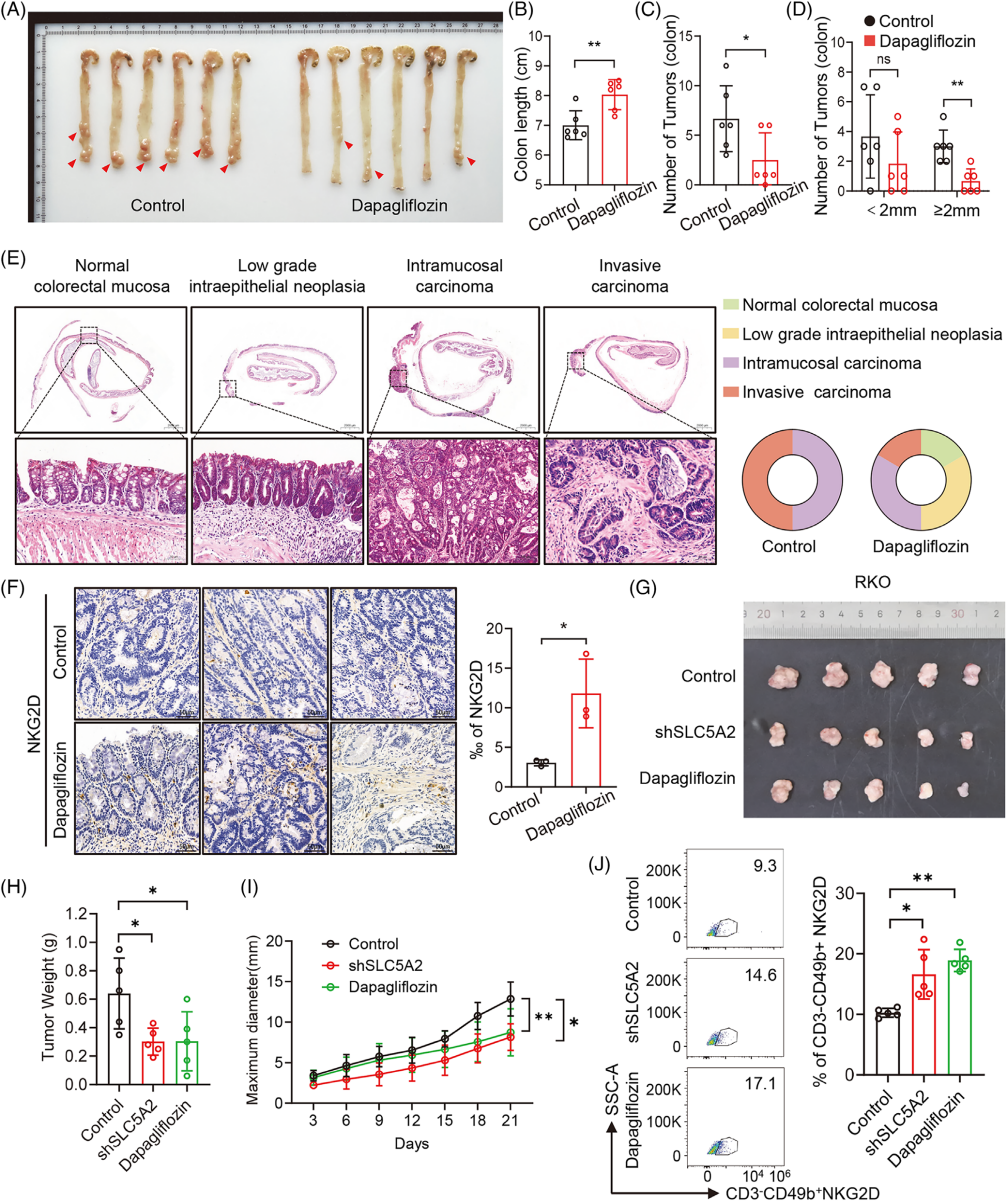
Dapagliflozin inhibits CRC tumourigenesis and progression. (A) Macroscopic images of colons from each group treated with AOM/DSS in C57BL/6 mice. (B–D) Statistical analysis of colon length, tumour number, and tumour load in mice treated with PBS or dapagliflozin. (E) Representative HE images of colorectal tumours at different stages and the proportions of each group (*n* = 6). (F) Representative images of NKG2D expression in tumour tissues from the PBS and dapagliflozin groups by IHC. (G) Effects of SLC5A2 silencing and dapagliflozin on subcutaneous tumours in nude mice. (H, I) Quantification of tumour weight (H) and tumour growth curve (I) (*n* = 5). (J) The percentages of NKG2D+ cells among CD3^−^CD49b^+^ NK cells from subcutaneous tumours were detected by flow cytometry.

Next, we applied the SLC5A2‐specific inhibitor dapagliflozin for the treatment of CRC and evaluated its antitumour effect. Following AOM/DSS induction in BALB/c mice, the treatment group received dapagliflozin via gavage, whereas the control group received PBS. Compared with the PBS‐treated group, dapagliflozin‐treated mice exhibited milder colon shortening, fewer and smaller tumours, decreased tumour infiltration, and increased infiltration of NK cells (Figure 4A–F). In the nude mouse subcutaneous xenograft model, SLC5A2 silencing and dapagliflozin treatment both reduced tumour weight and volume and increased infiltrating NK cell numbers (Figures 4G–J and S8C). These findings indicate that dapagliflozin has inhibitory effects on CRC tumourigenesis and the progression of existing tumours.

### 2.5 The SLC5A2–MICA/B–NKG2D axis has important clinical implications in CRC development

**FIGURE 5:**
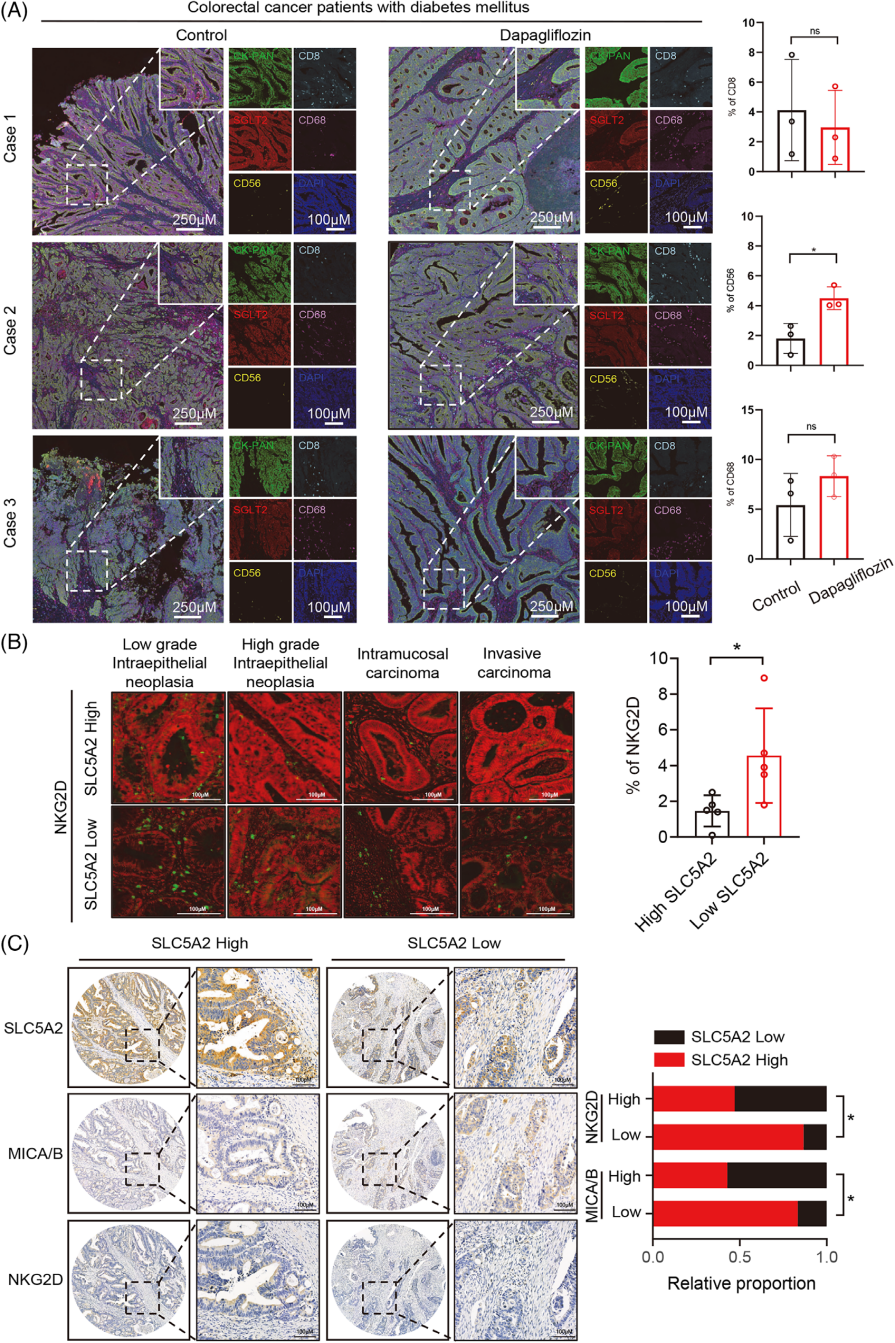
The SLC5A2–MICA/B–NKG2D axis has important clinical implications in CRC development. (A) The expression profiles of CK‐PAN, SLC5A2, CD56, CD8 and CD68 in CRC tissues of patients with CRC and diabetes were analysed by multiple IF. (B) The expression levels of NKG2D in tumours in the SLC5A2 low‐expression group and SLC5A2 high‐expression group at different stages of CRC were detected by multiple IHC. (C) IHC analysis of SLC5A2, MICA/B and NKG2D using tissue microarrays (left). Correlation analysis of SLC5A2 expression with MICA/B and NKG2D expression in CRC tissues (right).

We performed multiple immunofluorescence analysis on CRC tissues from diabetic patients with CRC who either received dapagliflozin treatment or not. Our findings revealed an increase in NK cell infiltration following dapagliflozin treatment, with no significant changes observed in T cells or macrophages (Figure 5A and Table S1). Furthermore, multiple histochemical analyses of postoperative tumour specimens from CRC patients showed that the infiltration density of NKG2D was lower in CRC tissues with high SLC5A2 expression than in those with low SLC5A2 expression within the same pathological grade (Figure 5B). Additionally, we further examined the associations between SLC5A2, MICA/B, and NKG2D in these clinical tissues. The results revealed that high SLC5A2 expression in CRC tissues was associated with low MICA/B and NKG2D expression, whereas low SLC5A2 expression was associated with the opposite correlation (Figure 5C). These results further confirm the important role of the SLC5A2–MICA/B–NKG2D axis in CRC development.

### 2.6 SLC5A2 is highly expressed in CRC and associated with poor patient survival

**FIGURE 6:**
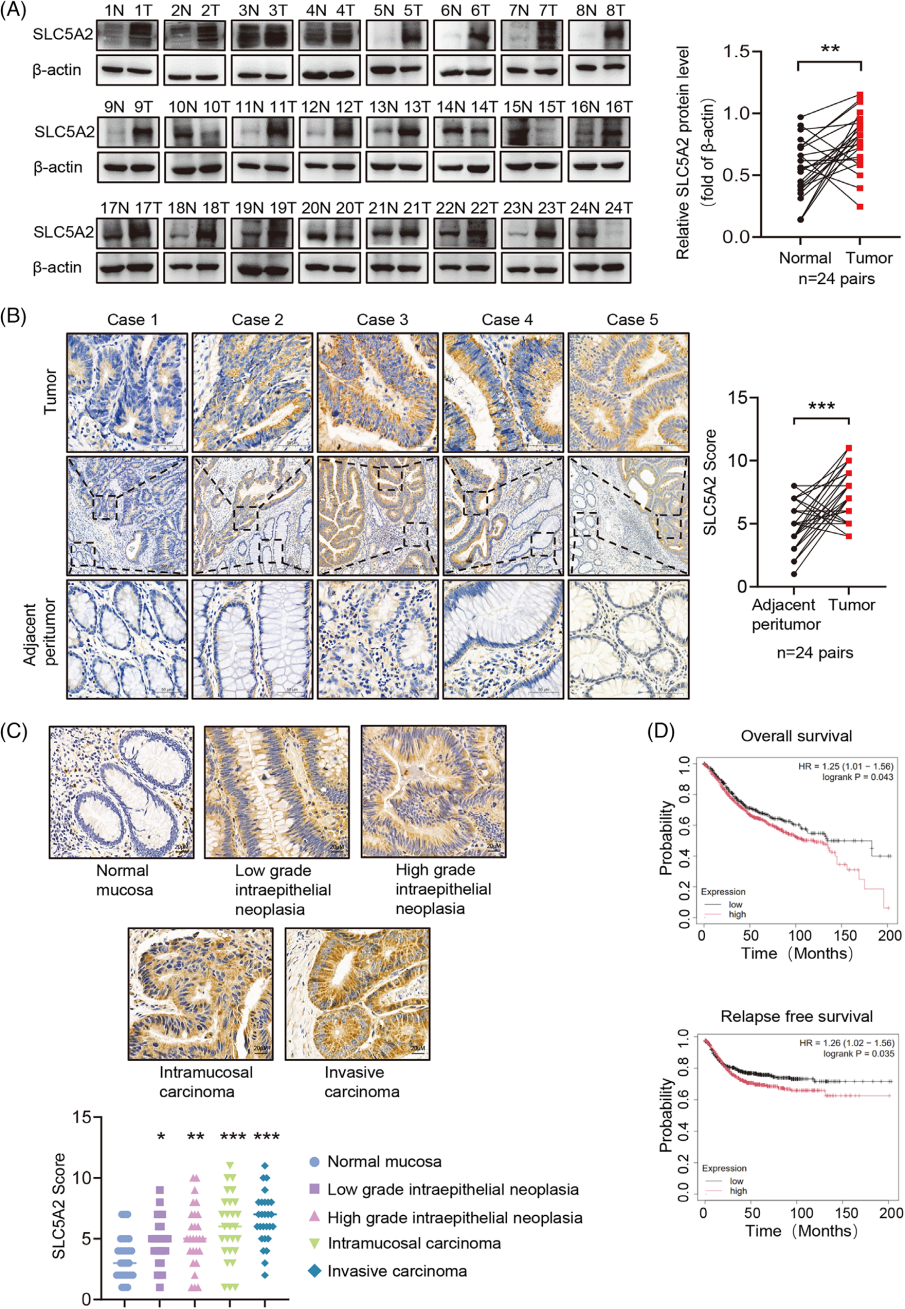
SLC5A2 is highly expressed and associated with poor survival in patients with CRC. (A) Western blot analysis of SLC5A2 protein expression in 24 paired CRC tumour tissues (T) and normal tissues (N). (B) Representative IHC images of SLC5A2 protein expression in 24 paired CRC tumour tissues and adjacent nontumour tissues. (C) IHC images of SLC5A2 in different clinical stages of CRC (*n* = 100). (D) Kaplan–Meier survival analyses of overall survival and relapse‐free survival based on SLC5A2 expression levels in patients with CRC using the Kaplan‒Meier plotter database.

To explore the expression and clinicopathological significance of SLC5A2 in patients with CRC, we detected SLC5A2 expression in human CRC samples. Western blot analysis revealed that SLC5A2 protein levels were significantly higher in CRC samples than in paired adjacent tissues (Figure 6A and Table S2), which was validated by IHC analysis of CRC and adjacent nontumour tissues (Figure 6B). Subsequent analysis demonstrated that SLC5A2 expression was upregulated in CRC tissues with disease progression of across different clinical stages (Figure 6C). Survival analysis further indicated that high SLC5A2 expression was associated with poor overall survival and recurrence‐free survival in CRC patients(Figure 6D). These findings demonstrate that SLC5A2 expression is upregulated in clinical CRC tissues and positively correlated with poor prognosis in patients with CRC, suggesting that SLC5A2 may be a potential therapeutic target for CRC.

## 3 DISCUSSION

The immune editing theory posits that tumour immune editing is a dynamic process consisting of three phases: elimination (tumour immune surveillance), equilibrium, and escape. Both adaptive and innate immune cells play pivotal roles in tumour immune surveillance, with NK cells acting as crucial effector cells.^24^ The capacity of NK cells to recognise and eliminate targets is central to immune surveillance, enabling them to eradicate tumour cells and virus‐infected cells in early stages of disease.^25^, ^26^ In malignancies such as CRC, gastric cancer, and melanoma, NK cell infiltration is correlated with favourable prognoses.^27^ Our research revealed that SLC5A2 can inhibit NK cell infiltration in CRC tissues to promote CRC progression, which has never been reported before.

The NK cell function is strictly regulated by surface activating and inhibitory receptors, and their activation is primarily triggered by the surface receptors NKG2D, NKp46 and NKp30.^28^ Studies have demonstrated that NKG2D‐activating receptors are critical for both innate and adaptive immune cells to distinguish tumour cells from normal cells.^29^, ^30^ Tumour cells characterised by a high mutation burden are likely to produce and present an increased number of neoantigens on MHC‐I molecules. This enhanced neoantigen presentationincreases the likelihood of tumour recognition, thereby stimulating immune cell activation and cytotoxicity.^31^ MICA/B are nonclassical MHC‐I molecules, and the NKG2D‐MICA/B receptor ligand system is the key link in tumour immune surveillance. The binding of MIC molecules on the tumour cell surface to NKG2D triggers the antitumour cytotoxic response of NK cells; and targeting this escape mechanism can restore antitumour immune responses.^29^, ^32^, ^33^ Therefore, the expression levels and stability of tumour surface antigens are key factors in immune surveillance and play important roles in tumour immune evasion. In this study, we demonstrated that SLC5A2 inhibits NK cell activation and tumouricidal function by preventing NKG2D from recognising MICA/B on the surface of CRC cells. This occurs through the promotion of MICA/B release from CRC cells, which may be particularly significant in precancerous lesions and early stages of CRC.

Solute carriers (SLCs) form a large family of membrane transporters that primarily responsible for facilitating the transmembrane transport of various substances, including ions, sugars, and nucleotides. Dysregulated SLCs expression is closely associated with metabolic diseases and tumourigenesis.^34^, ^35^ The SLC5A2 transporter is expressed in multiple tissues, including the kidney, intestine, pancreas, and heart. Research has indicated that SLC5A2 not only directly affects the metabolic stress of tumour cells through glucose uptake and oxidative phosphorylation,^36^, ^37^, ^38^ but also affects the intracellular calcium current in cardiomyocytes,^19^ and dapagliflozin, SLC5A2 inhibitor, is used in the clinical treatment of heart failure patients by reducing cardiac calcium load. But whether a similar mechanism exists in CRC cells remains unknown. Emerging evidence suggests that Calcium influx can directly increase the secretion of specific EV subsets.^39^ EVs from various cancer cells express the MIC family on their surfaces and that EVs expressing NKG2D ligands can induce NKG2D downregulation in NK cells and CD8^+^ T cells in vitro, thereby diminishing their cytotoxic function.^40^ From an electrophysiological perspective, this study revealed that the activation of the SLC5A2 channel generates an action potential that induces Ca^2+^ influx in CRC cells, subsequently promoting the release of MICA/B‐bearing EVs from tumours. This process potentially evades NK cell surveillance within the TME, providing novel insight into the mechanisms of tumour immune evasion. The safety and efficacy of dapagliflozin, a selective inhibitor of SLC5A2, have been widely verified as a clinical drug. Our investigations demonstrated that targeted inhibition of SLC5A2 can activate or restore the antitumour functions of NK cells, thereby effectively suppressing tumour growth, which provides a scientific rationale for the combined use of dapagliflozin with other therapies for CRC treatment.

However, some limitations should be considered when our findings are analysed. First, the MICA and MICB genes are located within the human HLA locus on chromosome 6. Although the mouse MHC locus lacks the MICA and MICB genes, the conserved ligand‐binding mechanism of NKG2D facilitates cross‐species recognition, thus permitting animal experiments using xenograft models.^41^, ^42^ Therefore, we used human CRC cell lines to establish a subcutaneous xenograft model in nude mice and verified their in vivo cytotoxic function by recognising the α1 and α2 domains of human MICA/B ligands via NK cells in nude mice. Notably, although this model can effectively detect NK cell function, it lacks adaptive immune components such as T cells and B cells and cannot fully simulate the complete immune microenvironment of the human body. Thus, the immunomodulatory effects observed in this study may not fully reflect the actual role of SLC5A2 inhibition in the context of an intact immune system. Second, GW4869 is a neutral sphingolipid inhibitor that modulates cell‐cell communication by inhibiting exosome biogenesis and secretion. Although it does not induce NK cell apoptosis or inhibit NK cell proliferation, its potential off‐target immune effects on NK cells remain unclear.^43^ In addition, NKG2D is expressed not only on NK cells but also on CD8+ T cells and γδT cells. In NK cells, NKG2D signalling alone is sufficient to trigger cytotoxic activity. However, in CD8+ T cells, NKG2D functions primarily as a costimulatory receptor to activate and enhance T‐cell receptor (TCR) signalling without directly mediating cytotoxicity; thus, the subsequent eradication of tumour cells relies on TCR‐mediated T‐cell activation.^44^, ^45^, ^46^ The effect of NKG2D on the surface of T cells does not conflict with our conclusions, as our research focused primarily on the cytotoxic effects mediated by NKG2D in NK cells. In this study, we utilised T‐cell‐deficient nude mice to evaluate the effects of a single NK‐cell factor in an animal model. Further investigations are needed to determine whether T cells and NK cells exert synergistic antitumour effects. Finally, all clinical samples in our current cohort are surgical resection specimens from treatment‐naive CRC patients who have not received immunotherapy. No public databases linking SLC5A2 expression with clinical responses to immune checkpoint inhibitors in CRC were identified after extensive literature and database screening, precluding a direct correlation analysis between SLC5A2 expression and immunotherapy efficacy at the present stage. This represents a clear limitation of our study, and investigating the predictive value of SLC5A2 for immunotherapy efficacy in immunotherapy‐treated patient cohorts will be a core direction of our subsequent research.

In conclusion, this study demonstrates that dapagliflozin can inhibit the action potential and Ca^2+^ influx in CRC cells by blocking the SLC5A2 ion channel. Additionally, it reduces the release of MICA/B from tumour cells, promotes NK cell infiltration in the TME, and enhances the immune surveillance and cytotoxic activity of NK cells against CRC cells. These findings suggest that dapagliflozin may inhibit CRC initiation and progression, thereby providing a promising foundation for its future application in CRC treatment.

## 4 MATERIALS AND METHODS

### 4.1 Animal models and AOM/DSS treatments

The construction and identification of SLC5A2 whole‐body gene knockout SD rats were performed by Saye Biotechnology Co., Ltd. Wild‐type SD rats and C57BL/6 mice were purchased from the Laboratory Animal Center of Southern Medical University. After adaptive feeding for 1 week, the rats were intraperitoneally injected with AOM solution (MP, Cat#0218397125, 15 mg/kg) 2 times. The mice were injected with AOM solution (MP, Cat#0218397125, 10 mg/kg) once, and DSS (YEASEN, Cat#60316ES60) solution was given at a drinking concentration of 2%. Each induction cycle consisted of 1 week of 2% DSS solution and 2 weeks of normal drinking water. During this period, the rats and mice presented bloody stools, weight loss, hair colour loss and other symptoms, indicating that the model was successful.

### 4.2 Subcutaneous xenograft model

The cells with good logarithmic growth were digested and prepared as a cell suspension (approximately 5×10^6^ cells). The suspension was implanted subcutaneously in the middle and posterior axillae of the 4–6 weeks mice. The treatment began when the tumour volume reached 100–200 mm^3^, and the experiment was terminated when the maximum tumour diameter reached 1.5 cm.

### 4.3 Cell culture and processing

Human normal colonic epithelial cells FHC, human CRC cell lines SW620, SW480, HCT15, Caco‐2, HCT116, RKO, and LoVo, and human embryonic kidney cell line cells HEK293T were purchased from the Cell Bank of Type Culture Collection of the Chinese Academy of Sciences (Shanghai, China) and passaged fewer than 30 times. All cell lines used in this study were mycoplasma‐negative and were authenticated by short tandem repeat (STR) profiling within four years. The shRNAs were constructed by Guangzhou Kinan Biotechnology Co., Ltd. The oeRNAs were constructed by Wuhan Miaoling Biotechnology Co., Ltd. CRC cells were treated in vitro in this study. The final concentrations of the chemicals used were as follows: 20 µM Dapagliflozin (Selleck, Cat#S1548); 50 µM α‐methylglucoside (Sigma, Cat#M9376‐100G); 10 µM Bay K 8644 (MCE, Cat#HY‐10588); 10 µM nifedipine (MCE, Cat#HY‐B0284); and 10 µM GW4869 (Selleck, Cat#S7609). The cells were cultured and transfected as described previously.^47^

### 4.4 Perforated patch‒clamp techniques

One day in advance, the cells were evenly spread on slides at a density of 30%–50%. The next day, each slide was immersed in extracellular solution (37°C) and opened with 95%:5% O_2_:CO_2_ mixed gas. The tip of a glass microelectrode was first inserted into freshly prepared nystatin (120–300 µg/mL), and then the freshly prepared intracellular liquid was injected into the electrode. After the cells were sealed to achieve high resistance, the perforated material formed pores in the cell membrane, and perforation patch‒clamp recording was performed. The membrane potential of the cells was recorded at 0 pA by clamping the membrane current.

### 4.5 Tumour‐infiltrating lymphocytes

Fresh tumour tissues were washed and cut into pieces and then digested with 200 U/mL DNA, 1 mg/mL collagenase, and  .5 mg/mL HA for 45 min. The digestion was terminated by adding complete medium, and the mixture was filtered and centrifuged. After screening, centrifugation, removal of the supernatant, and resuspension of the precipitate, the tumour‐infiltrating lymphocytes were separated by centrifugation with Percoll separation solution. Subsequent procedures were performed after resuspension in PBS.

### 4.6 Western blot

Western blot assays were performed according to standard protocols as previously described.^47^ The following antibodies were used: SLC5A2 (SANTA, Cat#sc‐393350), β‐actin (Proteintech, Cat#66009‐1‐Ig), MICA (Proteintech, Cat#66384‐1‐Ig), MICB (Abcam, Cat#ab300485), MICA + MICB (Abcam, Cat#ab224702), beta‐tubulin (Proteintech, Cat#10094‐1‐AP), ATP1A1 (Proteintech, Cat#14418‐1‐AP), CD63 (ABclonal, Cat#A5271), CD9 (ABclonal, Cat#A1703) and TSG101 (ABclonal, Cat#A2216).

### 4.7 Immunohistochemistry and multiplex immunofluorescence staining

Immunohistochemistry and immunofluorescence were performed according to the standard protocols described previously.^48^, ^49^ The following antibodies were used: SLC5A2 (SANTA, Cat#sc‐393350), SGLT2 (Abmart, Cat#PC20084), NKG2D (SANTA, Cat#sc‐515599), MICA + MICB (Abcam, Cat#ab224702), CD8 (Aifang Biological, Cat#AF20211), CD68 (Aifang Biological, Cat#AF20022), CD56 (Aifang Biological, Cat#14255‐1‐AP) and CK‐PAN (Aifang Biological, Cat#AF20164) were used.

### 4.8 Exosome isolation

Exosomes were isolated from cell culture supernatants using differential ultracentrifugation. Collected supernatants were centrifuged at 300 × *g* for 10 min to remove cells, followed by 2000 × *g* for 20 min to eliminate cell debris, and then 10 000 × *g* for 30 min to remove microvesicles and apoptotic bodies. The supernatant was then ultracentrifuged at 100 000 × *g* for 70 min to pellet exosomes. The pellet was washed with PBS and subjected to a second ultracentrifugation at 100 000 × *g* for 70 min.

### 4.9 Data availability

The gene expression data used in this study were obtained from the Gene Expression Omnibus (GEO) under accession number: GSE37892, GSE87211 and can be access at https://www.ncbi.nlm.nih.gov/gds; ABNORMAL_GLUCOSE_HOMEOSTASIS was obtained from the Molecular Signatures Database, available at https://www.gsea‐msigdb.org/gsea/index.jsp; Survival analysis data from https://kmplot.com/analysis/.

### 4.10 Statistical analysis

IBM SPSS Statistics 25 and GraphPad Prism 8.0 software were used for data processing and statistical analysis. All the experiments were independently repeated at least 3 times, but only the representative figure is displayed. For two‐group comparisons, an unpaired two‐tailed Student's *t*‐test was used for normally distributed data with equal variances, and Welch's *t*‐test was applied when variances were unequal. For multiple‐group comparisons, one‐way ANOVA was performed, followed by Tukey's post hoc test for equal variances or Dunnett's T3 post hoc test for unequal variances. For non‐normally distributed data, the Mann–Whitney U test was used for two‐group comparisons and the Kruskal–Wallis test with Dunn's post hoc test for multiple‐group comparisons. Tumour growth curves were analysed using two‐way repeated measures ANOVA with Tukey's multiple comparisons test. Survival curves were compared using the log‐rank test. A two‐tailed *p* < .05 was considered statistically significant (*p < .05, **p < .01, ***p < .001).

## AUTHOR CONTRIBUTIONS

**Jun Xiao**: Conceptualisation; methodology; investigation; data Curation; formal analysis; writing; visualisation. **Jianghua Wu**: Investigation; validation; data Curation. **Fengliu Deng**: Formal analysis; funding acquisition. **Chaoqun Liu**: Methodology; funding acquisition. **Yuanhang Chen**: Validation. **Ke Shen**: Clinical investigation. **Chuangyuan Wang**: Formal analysis; software. **Wandie Lin**: Methodology. **Weiwei Liu**: Software. **Ziyan Ning**: Resources. **Rui Zhou**: Conceptualisation; supervision. **Liang Zhao**: Conceptualisation; supervision; project administration; funding acquisition. All the authors approved the final version of the manuscript.

## CONFLICT OF INTEREST STATEMENT

The authors declare no conflicts of interest.

## ETHICS STATEMENT

All experiments involving patients were approved by the Ethics Committee of Shunde Hospital, Southern Medical University (Foshan, China) (Application No: KYLS20250753), and all aspects of the study comply with the Declaration of Helsinki. Informed consent was not required because the data were analysed anonymously. All animal procedures were approved by the Animal Care and Use Committee of Southern Medical University (SMUL202410026) and conducted under SPF conditions in accordance with approved protocols (Guangzhou, China).

## PATIENT AND PUBLIC INVOLVEMENT

Patients and/or the public were not involved in the design, or conduct, or reporting, or dissemination plans of this research.

## Supporting information



Supporting information

Supporting information

## Data Availability

The datasets generated and/or analysed during the current study are not publicly available but are available from the corresponding author upon reasonable request.
