# Supplementary material for: Targeting SLC5A2 suppresses colorectal tumour development by enhancing NK cell activity through extracellular vesicle‐dependent MICA/B signalling
Source: Clin Transl Med. 2026 Apr 12;16(4):e70657. doi: 10.1002/ctm2.70657 (PMC13071181; doi:10.1002/ctm2.70657)
Supplement: Supplementary file 1 — Supporting information [file CTM2-16-e70657-s002.docx]

**Supplementary Tables**

**Table S1.** Clinical information of patients in multiplex immunofluorescence.

| **Gender** | **Age** | **Type of tumor** | **TNM** | **Drugs** |
| --- | --- | --- | --- | --- |
| Male | 64 | Adenocarcinoma | T4N2M1 | N/A |
| Female | 84 | Adenocarcinoma | T3N2aM0 | N/A |
| Male | 65 | Adenocarcinoma | T3N0M0 | N/A |
| Female | 68 | Adenocarcinoma | T4bN2bM0 | Dapagliflozin |
| Male | 67 | Adenocarcinoma | T2N0Mx | Dapagliflozin |
| Male | 59 | Adenocarcinoma | T1N0M0 | Dapagliflozin |

**Table S2.** Clinical information of patients in 24 paired CRC tumour tissues.

| **Gender** | **Age** | **Type of tumor** | **depth of invasion** |
| --- | --- | --- | --- |
| Male | 64 | Adenocarcinoma | submucosa |
| Male | 77 | Adenocarcinoma | submucosa |
| Female | 65 | Adenocarcinoma | submucosa |
| Male | 76 | Adenocarcinoma | submucosa |
| Male | 73 | Adenocarcinoma | muscularis mucosae |
| Female | 67 | Adenocarcinoma | muscularis mucosae |
| Male | 76 | Adenocarcinoma | muscularis mucosae |
| Female | 72 | Adenocarcinoma | Full-thickness intestinal wall |
| Male | 68 | Adenocarcinoma | Full-thickness intestinal wall |
| Male | 66 | Adenocarcinoma | Full-thickness intestinal wall |
| Male | 77 | Adenocarcinoma | Full-thickness intestinal wall |
| Male | 75 | Adenocarcinoma | Full-thickness intestinal wall |
| Male | 90 | Adenocarcinoma | Full-thickness intestinal wall |
| Male | 65 | Adenocarcinoma | Full-thickness intestinal wall |
| Male | 76 | Adenocarcinoma | Full-thickness intestinal wall |
| Male | 72 | Adenocarcinoma | Full-thickness intestinal wall |
| Female | 70 | Adenocarcinoma | Full-thickness intestinal wall |
| Male | 72 | Adenocarcinoma | Full-thickness intestinal wall |
| Male | 67 | Adenocarcinoma | Full-thickness intestinal wall |
| Male | 75 | Adenocarcinoma | Full-thickness intestinal wall |
| Male | 53 | Adenocarcinoma | Full-thickness intestinal wall |
| Female | 41 | Adenocarcinoma | Full-thickness intestinal wall |
| Male | 68 | Adenocarcinoma | Full-thickness intestinal wall |
| Male | 61 | Adenocarcinoma | Full-thickness intestinal wall |
