# Supplementary material for: Targeting SLC5A2 suppresses colorectal tumour development by enhancing NK cell activity through extracellular vesicle‐dependent MICA/B signalling
Source: Clin Transl Med. 2026 Apr 12;16(4):e70657. doi: 10.1002/ctm2.70657 (PMC13071181; doi:10.1002/ctm2.70657)
Supplement: Supplementary file 2 — Supporting information [file CTM2-16-e70657-s001.docx]

**Supplementary Figures**


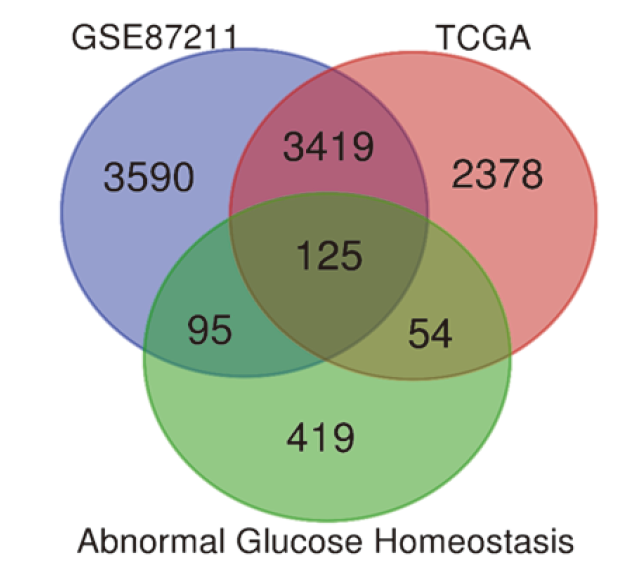


**Figure S1.** Intersection operation of TCGA data set and GSEA data set to obtain Venn diagram.


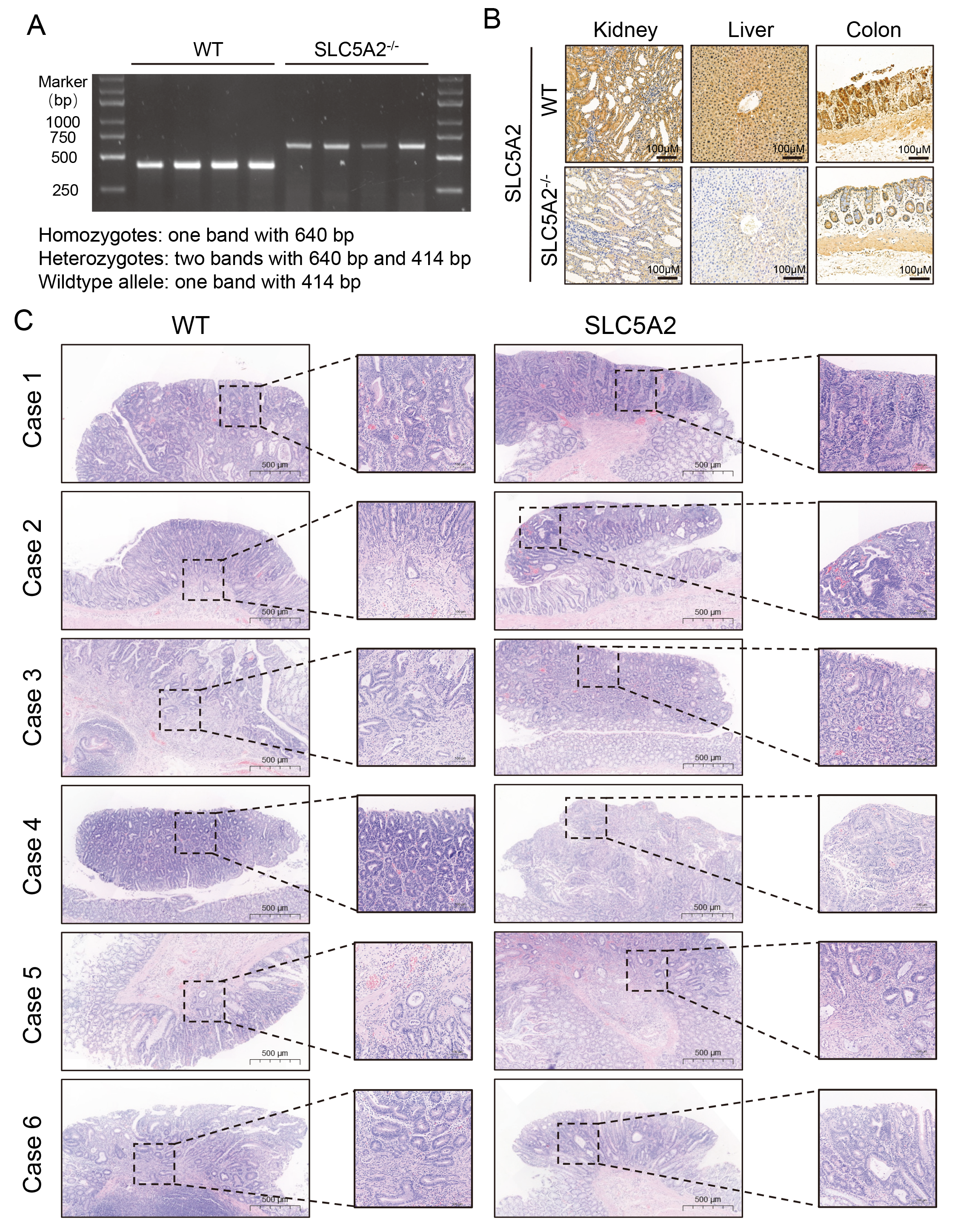


**Figure S2.** (A) Identification results of SLC5A2^-/-^SD rat construction.（B）IHC of SLC5A2 in the kidneys, livers and colons of the WT group and the SLC5A2^-/-^ group. (C) HE diagnostic images of all animals in the WT group and the SLC5A2^-/-^ group.


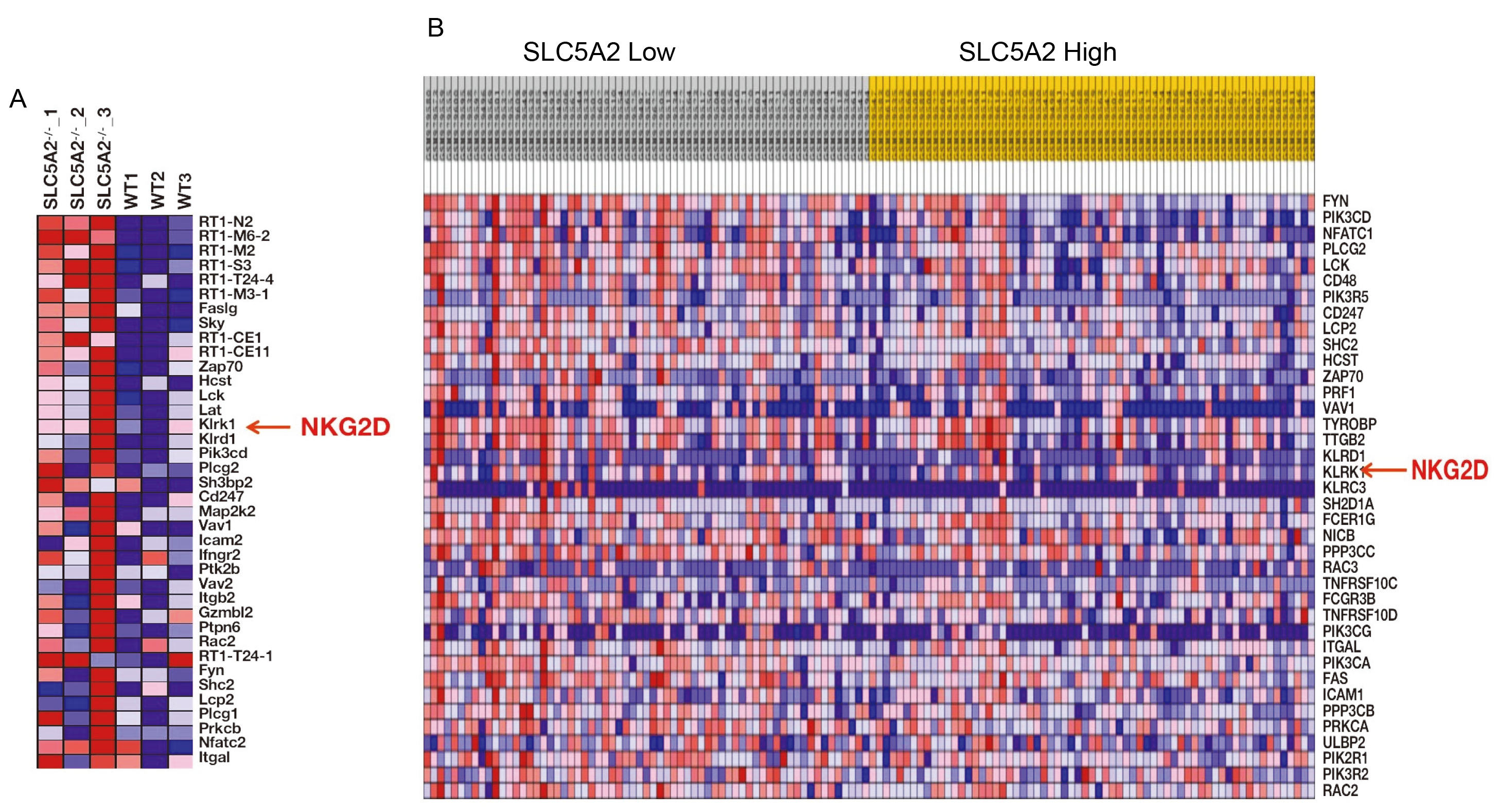


**Figure S3.** GSEA enrichment analysis of rat AOM/ DSS-induced CRC (A) and human CRC (B) core genes in NK cell-mediated cytotoxicity.


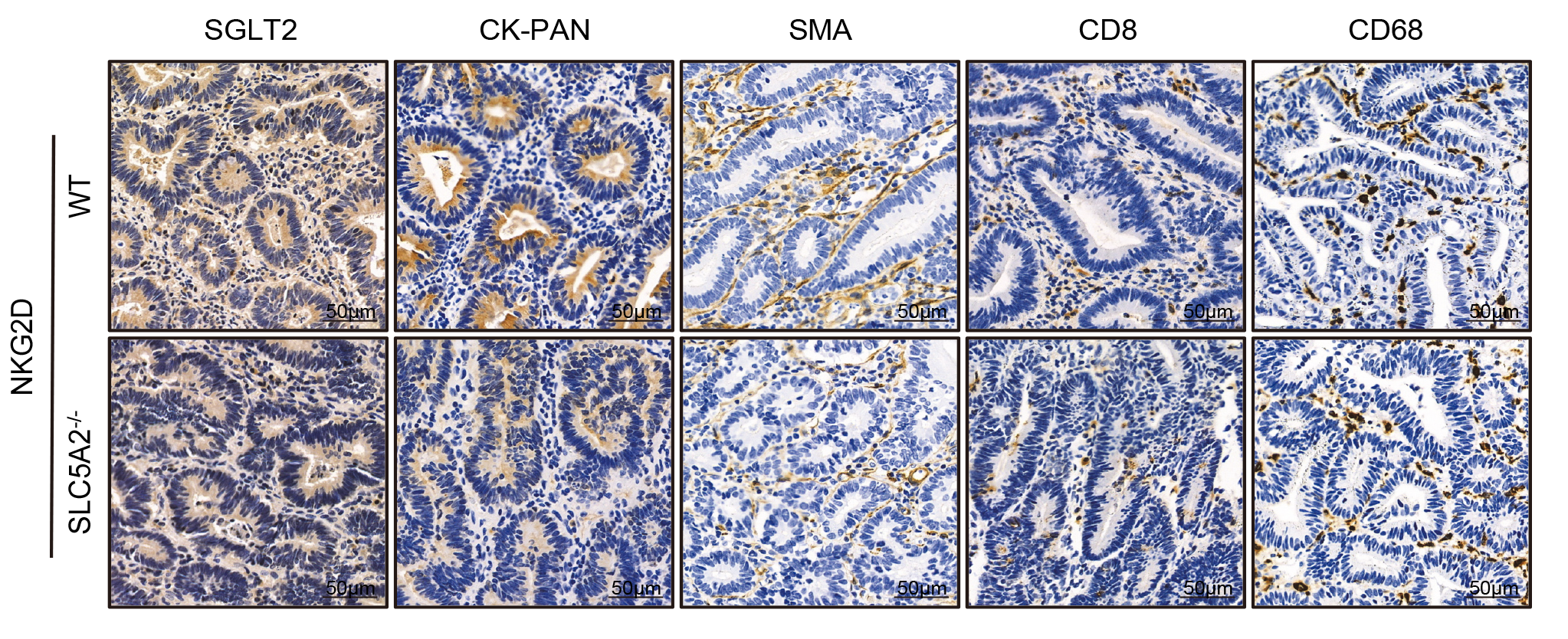


**Figure S4.** IHC analysis of SGLT2, CK-PAN, SMA, CD8 and CD68 in CRC tumors of WT group and SLC5A2^-/-^ group.


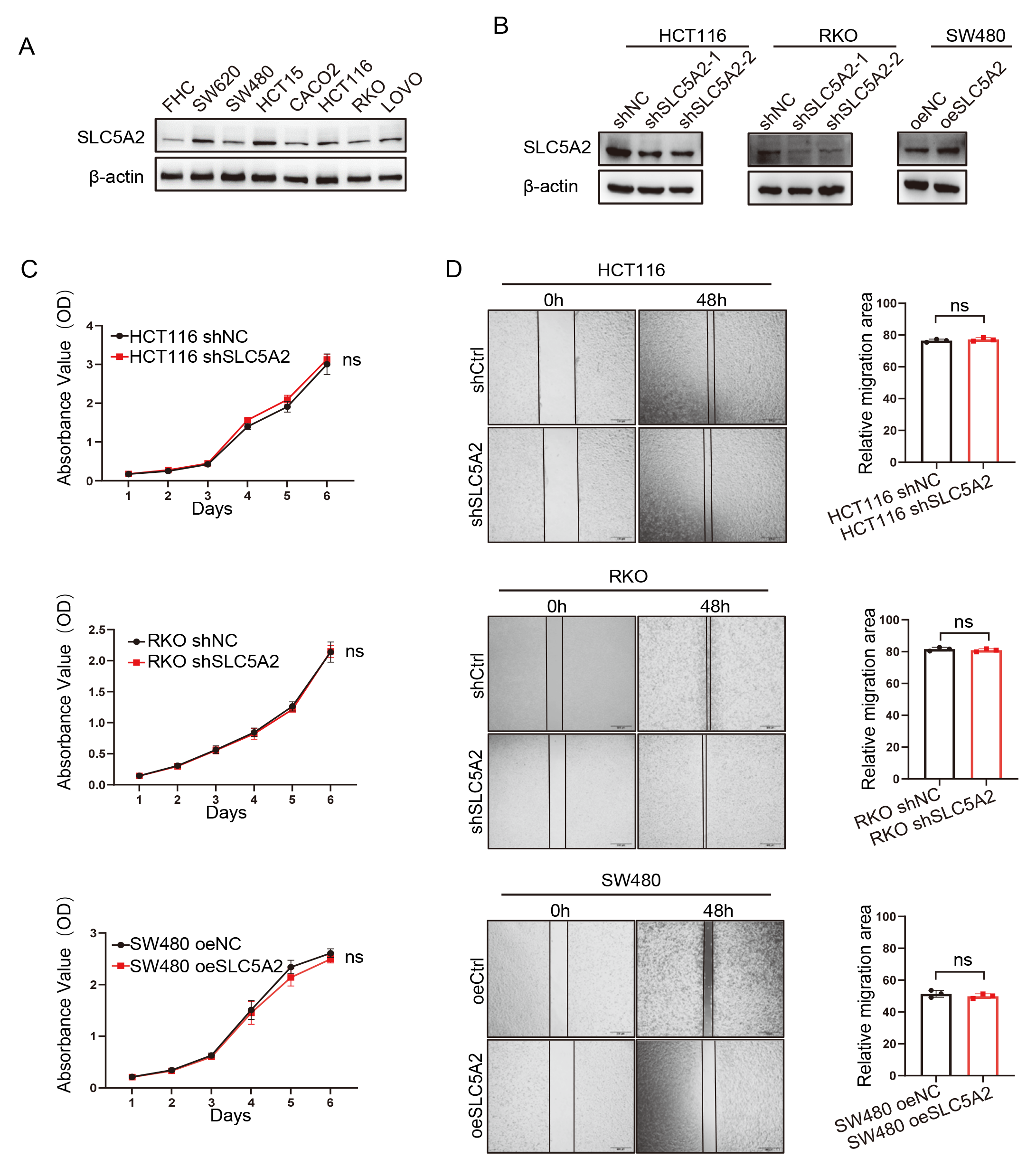


**Figure S5.** (A) Western blot analysis of SLC5A2 expression abundance in CRC cell lines. (B) Validation of the expression efficiency of stably silenced and overexpressed SLC5A2 human CRC cell lines. (C) The effect of SLC5A2 on the proliferation of HCT116, RKO and 480 cells was detected by CCK8. (D) The effect of SLC5A2 on the migration function of HCT116, RKO and 480 cells was detected by scratch test.


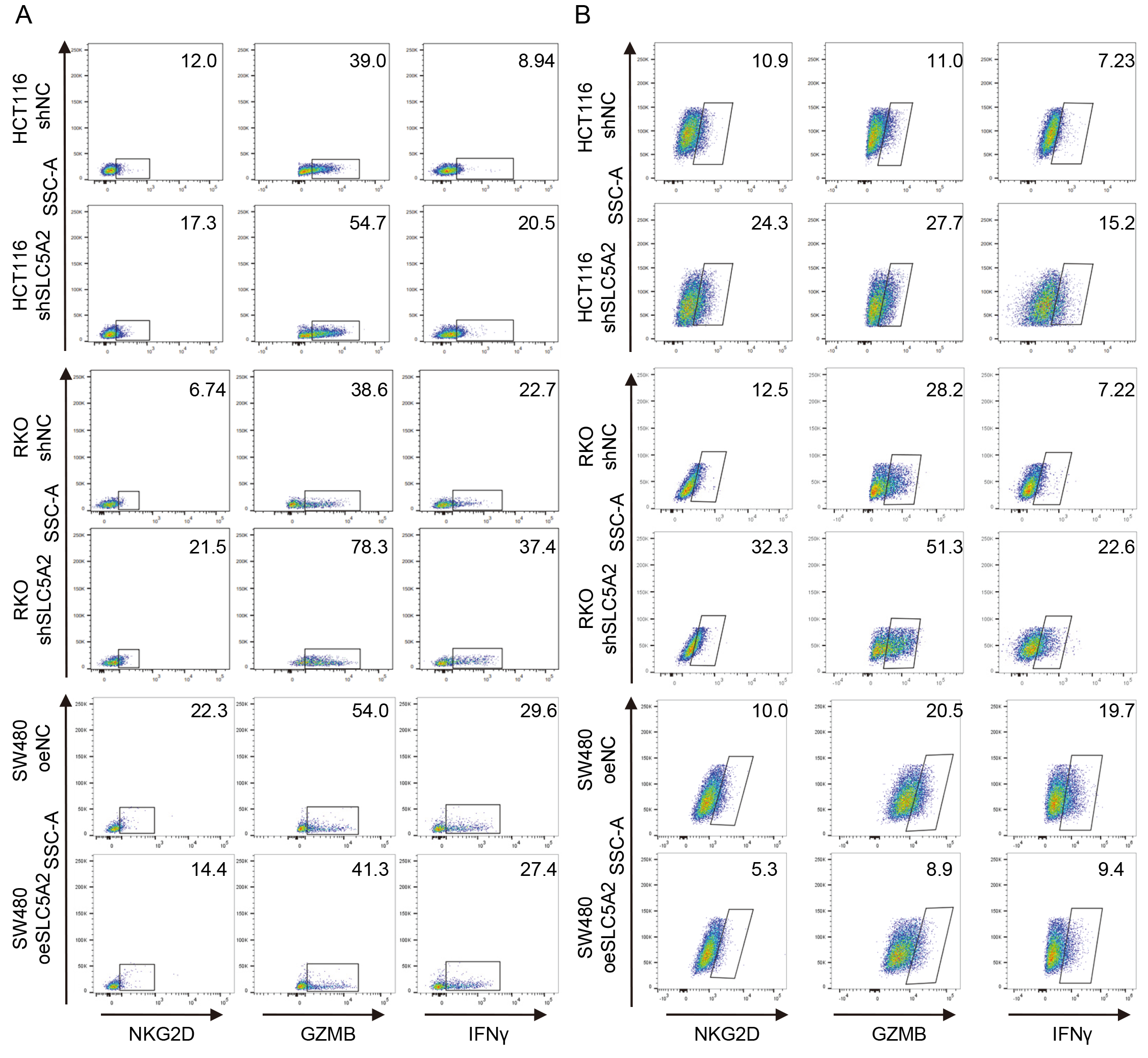


**Figure S6.** The NKG2D, IFN-γ and GZMB levels of NK cells were detected by flow cytometry after CRC cells were co-culture with PBMC (A) and NK92 cells (B).


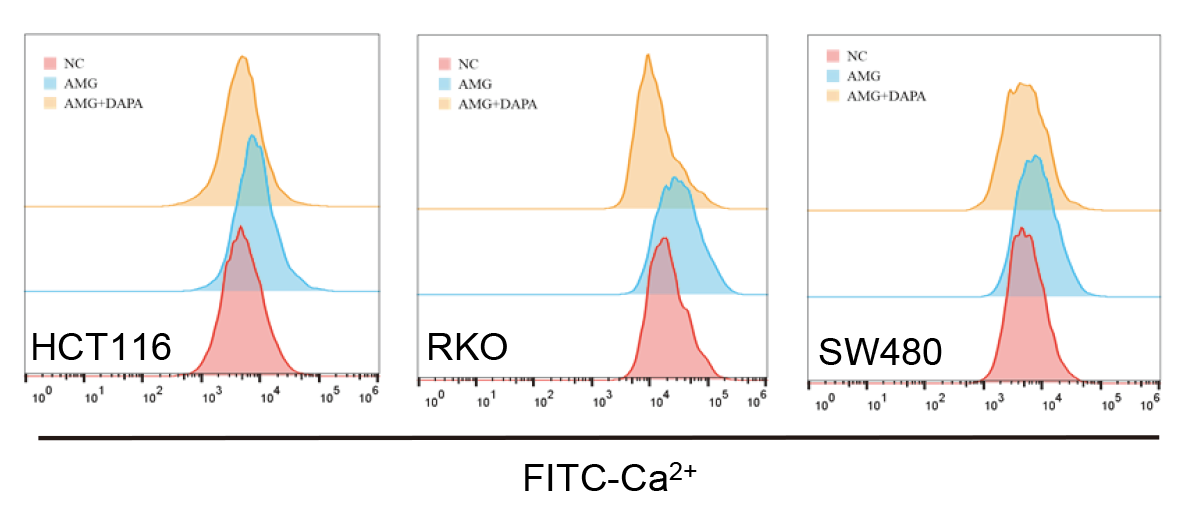


**Figure S7.** Flow cytometry was used to detect the effect of SLC5A2 agonists and inhibitors on intracellular calcium in HCT116, RKO and SW480 cells.


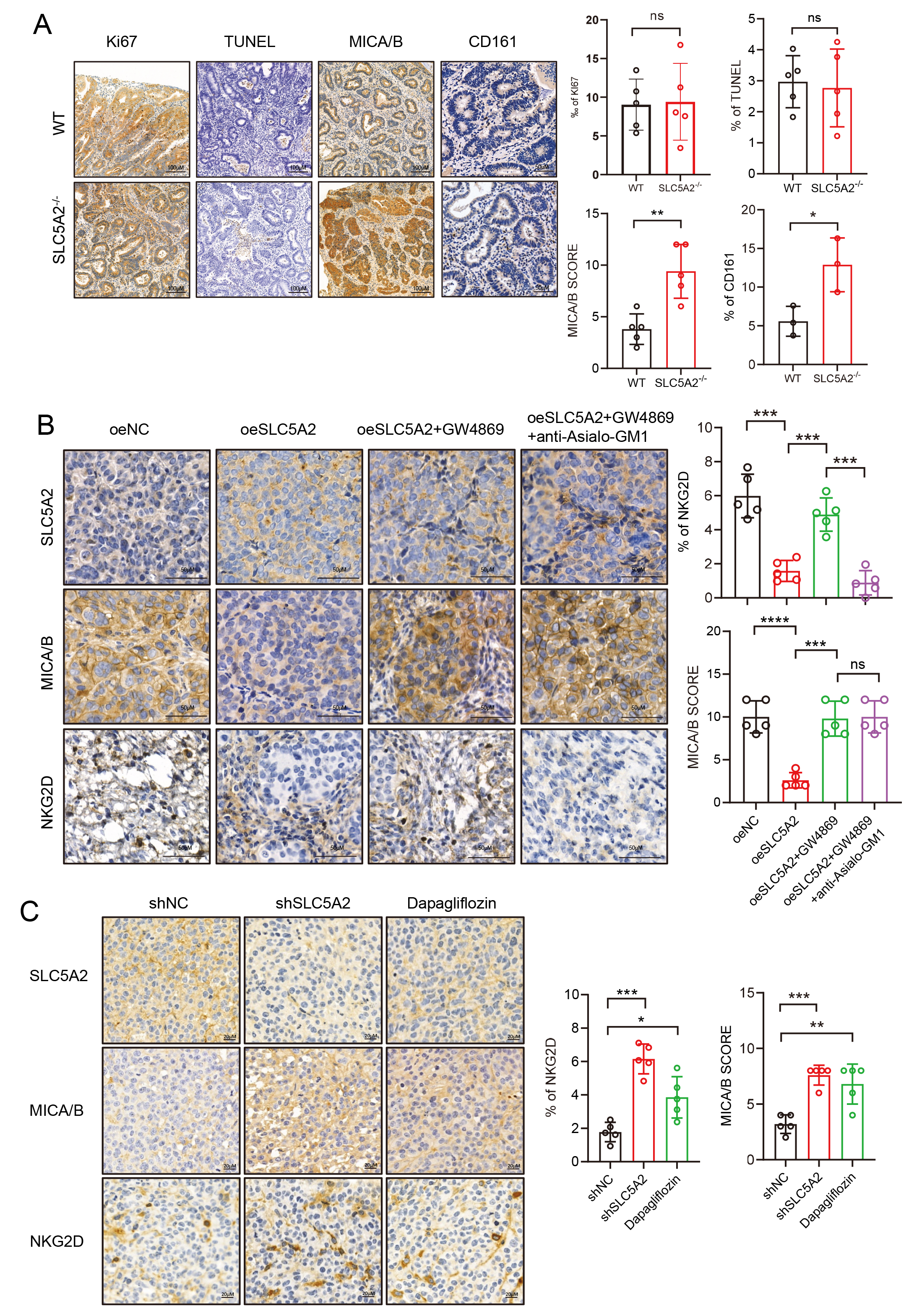


**Figure S8.** (A) IHC analysis of Ki67, Tunel, MICA/B and CD161 in CRC tumors of WT group and SLC5A2-/- group. (B-C) IHC was performed to detect the expression of SLC5A2, MICA/B and NKG2D in the indicated subcutaneous tumors in nude mice.
